# Nucleosome positioning is unaltered at *MLH1* splice site mutations in cells derived from Lynch syndrome patients

**DOI:** 10.1186/s13148-014-0032-6

**Published:** 2014-12-13

**Authors:** Mathew A Sloane, Luke B Hesson, Andrea C Nunez, Bryony A Thompson, Robyn L Ward

**Affiliations:** Adult Cancer Program, Lowy Cancer Research Centre and Prince of Wales Clinical School, University of New South Wales, Sydney, NSW Australia; Department of Genetics and Computational Biology, QIMR Berghofer Medical Research Institute, Brisbane, QLD Australia

**Keywords:** Lynch syndrome, Colorectal cancer, Nucleosome, Splice site, Splicing, Acceptor, Donor, Exon

## Abstract

**Background:**

Splicing is more efficient when coupled with transcription and it has been proposed that nucleosomes enriched in exons are important for splice site recognition. Lynch syndrome is a familial cancer syndrome that can be caused by the autosomal dominant inheritance of splice site mutations in the MutL homolog 1 (*MLH1*) gene. To better understand the role of nucleosomes in splicing, we used *MLH1* splice site mutations in Lynch syndrome cases as a model to investigate if abnormal splicing was associated with altered nucleosome positioning at exon-intron boundaries.

**Findings:**

Nucleosome Occupancy and Methylome sequencing (NOMe-seq) was used to determine the allele-specific positioning of nucleosomes around heterozygous splice site mutations in lymphoblastoid cells lines (LCLs) derived from six Lynch syndrome patients. These mutations were previously shown to cause exon skipping in five of the six patients. Allele-specific high-resolution nucleosome mapping across exons and exon-intron boundaries revealed high levels of nucleosomes across all regions examined. Alleles containing donor or acceptor splice site mutations showed no consistent alteration in nucleosome positioning or occupancy.

**Conclusion:**

Nucleosomes were enriched at *MLH1* exons in LCLs derived from Lynch syndrome patients, and in this model system the positioning of nucleosomes was unaltered at exon-intron boundaries containing splice site mutations. Thus, these splice site mutations alone do not significantly change the local organisation of nucleosomes.

## Findings

### Background

Splicing removes introns from a longer pre-cursor RNA molecule to produce a final processed mRNA. Splice donor and acceptor sites located at the junctions between exons and introns contain conserved sequence elements that are necessary for splicing [1]. Although *in vitro* splicing can occur, splicing is more efficient when coupled with transcription [2], suggesting that factors additional to the nucleic acid sequence are important for splice site recognition. It has been proposed that the positioning of nucleosomes at exons aids in splice-site recognition [3,4].

Nucleosome positioning describes the precise location of a given nucleosome, whereas nucleosome occupancy refers to the proportion of molecules bearing a nucleosome at a specific location, at any given instant [5]. It is hypothesised that nucleosomes positioned within exons, especially those with weak splice sites, cause RNA Polymerase II (RNAPII) to pause, enabling an interaction with the spliceosome and more efficient splicing [3]. In support of this, nucleosome occupancy is enriched across exons [3,4], which have a high GC content that favours nucleosome assembly [3,6], and the average size of an exon within the body of a human gene is 151 bp, that is similar in length to nucleosomal DNA (approximately 147 bp) [7]. Splicing factors associate with the C-terminal domain of RNA Polymerase II (RNAPII) [8], while the histone modification H3K36me3 is enriched in exons [3,9], where it may act as a scaffold to recruit splicing factors [9]. Taken together this indicates there is interplay between nucleosomes, the splicing machinery and the DNA sequence.

Lynch syndrome is an autosomal dominant familial cancer syndrome characterised by early onset colorectal, endometrial and other cancers [10,11]. It is most commonly caused by the inheritance of heterozygous loss-of-function mutations, including splice site mutations, in the DNA mismatch repair (MMR) genes *MLH1* and *MSH2* [12]. In this study we used *MLH1* splice site mutations in Lynch syndrome as a model to better understand the role of nucleosomes in splicing. We investigated cells from Lynch syndrome patients with splice site mutations to determine whether splicing aberrations were associated with altered nucleosome positioning at exon-intron boundaries.

## Materials and methods

### Identification of *MLH1* splice site mutations

Bioinformatic analysis and *in vitro* assays previously showed that genetic mutations at exon-intron boundaries in the *MLH1* gene generate aberrantly spliced transcripts (see Table 1). All mutations were classified as pathogenic (Class 5) according to the International Society for Gastrointestinal Hereditary Tumours Variant Interpretation Committee (InSiGHT VIC) [12,13].

**Table 1:** **Molecular features of** ***MLH1*** **splice site mutations in six individuals with Lynch syndrome**

**Mutation**	**Mutation location (GRC37, Feb 2009)**	**Affected splice site**	**Nucleosome occlusion of splice site**		**Splicing error** [13]
			**Allele**	**Proportion of alleles**	
c.588 + 1G > T	Chr. 3: 37,053,354	Donor	Wild-type (G)	19/20	Exon 7 skipping (r.546_588del)
	First bp of intron 7		Mutant (T)	21/22	
		Acceptor	Homozygous	36/42	
c.589-2A > G	Chr. 3: 37,053,500	Acceptor	Wild-type (A)	17/20	4 bp deletion in cDNA (r.589_592del)
	2nd last bp of intron 7		Mutant (G)	19/22	
c.790 + 1G > T	Chr. 3: 37,056,036	Donor	Wild-type (G)	33/34	Unknown
	1st bp of intron 9		Mutant (T)	21/24	
c.791-1G > C	Chr. 3: 37,058,996	Acceptor	Wild-type (G)	30/30	Exon 10 skipping (r.791_884del)
	Last bp of intron 9		Mutant (C)	12/18	
c.884G > A	Chr. 3: 37,059,090	Donor	Wild-type (G)	42/42	Exon 10 skipping (r.791_884del)
	Last bp of exon 10		Mutant (A)	24/30	
c.1559-2A > T	Chr. 3: 37,081,675	Acceptor	Wild-type (A)	17/20	Two aberrant transcripts (Exon 14 skipping or exon 14 and 15 skipping – r.[1559_1667del, 1559_1731del])
	Second last bp of intron 13		Mutant (T)	16/16	

### Cell culture

LCLs were established from patient blood by transformation with Epstein-Barr Virus (as described previously) [13], and cultured in RPMI with 10% fetal bovine serum (Gibco, Life Technologies) at 37°C in 5% CO_2_. Lymphocytes for transformation from five patients (c.588 + 1G > T, c.589-2A > G, c.791-1G > C, c.884G > A and c.1559-2A > T) were obtained from the Australasian Colorectal Cancer Family Registry (ACCFR) [14]. Lymphocytes from the patient with the c.790 + 1G > T mutation were obtained from the MCO collection [15].

### NOMe-seq

NOMe-seq was performed as described previously [16]. Briefly, intact nuclei were treated with 200 to 300 U GpC methyltransferase M.*Cvi*Pl and 160 to 320 μM S-adenosylmethionine for 15 min at 37°C followed by termination of the reaction with an equal volume of 20 mM Tris HCl pH 7.9, 600 mM NaCl, 1% (w/v) SDS and 10 mM EDTA. DNA was extracted using phenol chloroform followed by ethanol precipitation and bisulfite modified using the EZ DNA Methylation-Gold™ Kit (Zymo Research). Regions incorporating the splice site mutation in each patient were amplified from 40 ng of bisulfite treated DNA using a nested PCR with the primers and annealing temperatures described in Table 2. Single molecule sequencing of PCR amplicons was performed as described previously [17]. Wild-type and mutant alleles were distinguished using the splice site sequence alteration. The M.*Cvi*PI enzyme methylates accessible DNA at GpC sites, whereas nucleosome bound DNA is inaccessible and remains refractory to GpC methylation. Regions of M.*Cvi*PI inaccessibility of ≥150 bp (the length of DNA wrapped around a single nucleosome) within a single molecule were considered to represent regions of nucleosome occupancy. In addition, NOMe-seq retains the endogenous methylation status of the DNA allowing nucleosome positions and DNA methylation to be determined on each molecule.

**Table 2:** **NOMe-seq primer sequences and amplification conditions**

**Region**	**NOMe-seq primers**	**Annealing (°C)**
c.588 + 1G > T	F:TTGATATTTAGTGTGTGTTTTTGGYAAT	F/R = 54°C
	R:CACATAATATCTTAAAAAATTCCAAAATAATA	F/RN = 56°C
	RN:ATACCRACTAACARCATTTCCAAAAATAA	
c.589-2A > G	F:TTAGGTATTTAGTATATAATGYAGG	F/R = 51°C
	R:CACTATAAATATTTTCAAAACTAAAACCTTA	F/RN = 52°C
	RN:CACAAAATCTAAAAAATTACATACACCTAA	
c.790 + 1G > T	F:TAGGYATAGGAGGATTTTAAATGGATTAAGTT	F/R = 52°C
	R:CAATTTCTTTAATAACAATRCCTATACCTAAA	F/RN = 52°C
	RN:TTACTCRTAAAAACTCTAACACCATCAA	
c.791-1G > C	F:GATGTGATGTGYATATTATTATAGAAATGTT	F/R = 55°C
	R:TATCARCACCTCCTAATAAAATGAARCATA	F/RN = 56°C
	RN:ATCCTTTTRCCAATAATATATAAAATTCACTCTA	
c.884G > A	F:GGATGTGATGTGYATATTATTATAGAAATGTT	F/R = 58°C
	R:CTATTATARCTTCCCAACTAACCCCARCAA	F/RN = 58°C
	RN:CTACAARCTATCARCACCTCCTAATAA	
c.1559-2A > T	F:TATTAGGAGGYTTAATTTAGGYTTTTTTGYTTAT	F/R = 58°C
	R:ACCCTCACCACCTAATTCACAACATTTATAA	F/RN = 57°C
	RN:ACTAARCAACTACCAAAAACTAARCTTCTTA	
*HSPA5*	F: GAGAAGAAAAAGTTTAGATTTTATA	F/R = 56°C
	R: AAACACCCCAATAAATCAATC	

## Results

**Figure 1:**
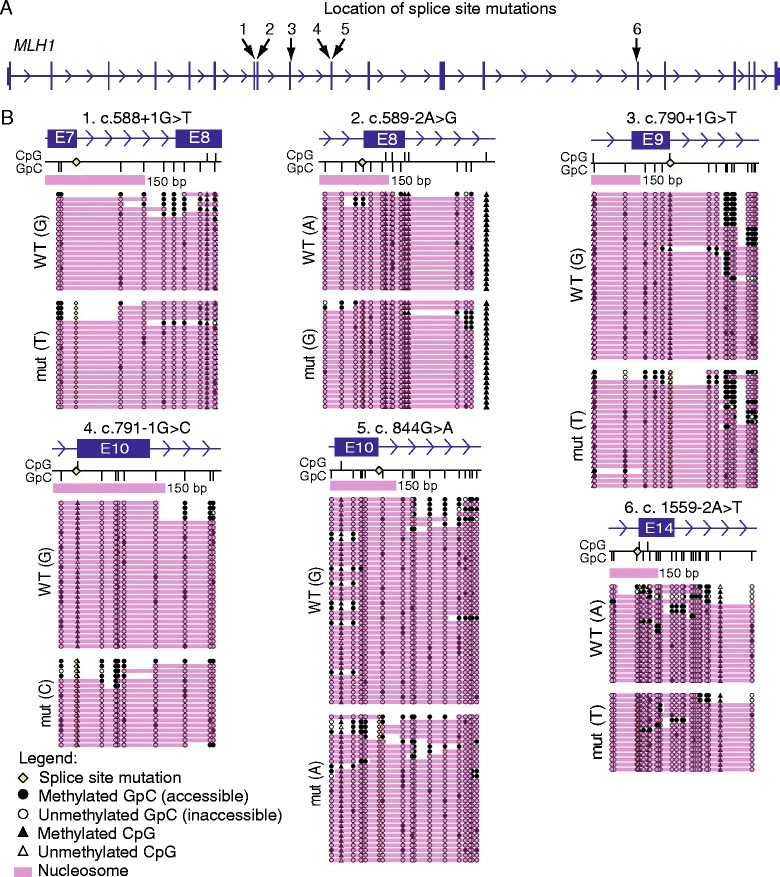
**Allele-specific positioning of nucleosomes at** ***MLH1*** **variants. (A)** The *MLH1* gene with arrows showing the location of the splice site mutations investigated in the LCLs derived from the six patients. The numbers correspond to the NOMe-seq assays shown in panel B (exon, vertical bar; intron, horizontal bar with arrows). **(B)** Each panel shows the nucleosome occupancy on the wild-type allele (upper) and the allele harbouring the indicated splice site mutation (lower). Blue box = exon; blue arrows = intron and direction of transcription; yellow diamond = location of splice site mutation; vertical black bars below line = GpC sites; vertical black bars above line = CpG sites; the pink bar represents a single nucleosome (150 bp, drawn to scale); pink shading indicates the location of nucleosomes on individual DNA molecules as determined by GpC methyltransferase inaccessibility; black circles = methylated GpC/accessible to M.*Cvi*PI; white circles = unmethylated GpC/inaccessible to M.*Cvi*PI. CpG sites are depicted by triangles. Black triangle = methylated CpG; white triangle = unmethylated CpG.

**Figure 2:**
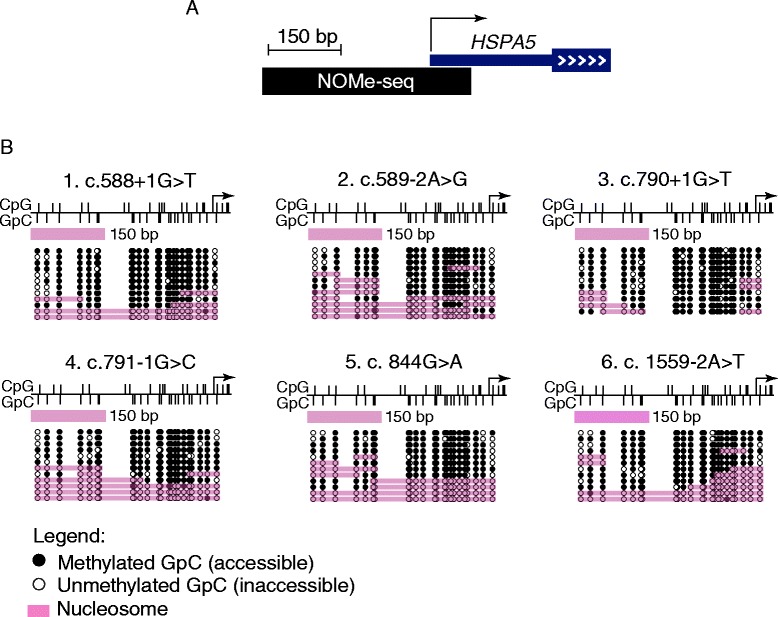
**Nucleosome occupancy and endogenous CpG methylation at the** ***HSPA5*** **gene. (A)** Schematic showing the location of the NOMe-seq assay (black bar) in relation to the *HSPA5* promoter. The arrow represents the transcriptional start site. **(B)** NOMe-seq was used to map the position of nucleosomes on individual DNA molecules at the *HSPA5* gene in LCLs derived from the six individuals with splice site mutations in *MLH1*. Vertical black bars below line = GpC sites; vertical black bars above line = CpG sites; pink bars = the location of nucleosomes on individual DNA molecules as determined by GpC methyltransferase inaccessibility; black circles = methylated GpC/accessible to M.*Cvi*PI; white circles = unmethylated GpC/inaccessible to M.*Cvi*PI. All endogenous CpG sites were unmethylated and triangles are not shown to provide better clarity of GpC sites.

We determined the positioning of nucleosomes across exons 7, 8, 9, 10 or 14 of the *MLH1* gene in LCLs derived from six Lynch syndrome patients. Splice site mutations within or near these exons affected splice donor or acceptor sites (Table 1). NOMe-seq and single molecule sequencing allowed us to distinguish mutant from wild-type alleles using the splice site sequence mutation. At all sites analysed the majority of molecules were inaccessible at GpC sites and methylated at CpG sites (Figure 1). NOMe-seq analysis of the control gene *HSPA5*, a constitutively active gene that maintains a nucleosome-depleted region at the transcription start site [18], showed that the CpG island was unmethylated (data not shown) and confirmed that GpC inaccessibility was due to nucleosome occupancy rather than incomplete M.*Cvi*PI treatment (Figure 2). This shows that the majority of molecules at the sites analysed are occupied by nucleosomes in these cells.

Separation of alleles by presence or absence of the splice site mutation showed no overall difference in either nucleosome positioning or occupancy between wild-type and mutant alleles (Figure 1 and Table 1). In the six splice site mutations analysed, three were located within donor splice sites and three were located within acceptor splice sites (Table 1). Though some difference in precise positioning between mutant and wild-type alleles was observed across some exons (for example, exons 7 and 10, Figure 1B) this was confined to a small subset of molecules, rather than the majority of molecules as would be expected if the mutations affected nucleosome positioning. Our study therefore shows that nucleosome occupancy is unaltered at exon-intron boundaries containing these splice site mutations.

## Discussion

In this study, we show that nucleosome positioning is unaltered at exon-intron boundaries containing splice site mutations. This finding suggests that the positioning of nucleosomes at exon-intron boundaries containing splice site mutations does not play a role in the splicing defect.

NOMe-seq has been used to measure nucleosome occupancy at gene promoters, CTCF binding sites [19] and regulatory elements [20] but this is the first study that has utilised the technique to measure nucleosome occupancy at splice sites. NOMe-seq previously showed that the active CpG island (CGI) promoter of *HSPA5* has a nucleosome-depleted region (NDR) upstream of the transcriptional start site [19]. This region was included as a control in each assay, and in all cases the NDR was present. This indicated that the absence of GpC methylation observed in *MLH1* was caused by nucleosome occlusion, not an artefact caused by a defective M.*Cvi*PI enzyme. In addition, CpG methylation was absent from the *HSPA5* NDR which is a feature of active CGI promoters [21]. Finally, gene body methylation is a feature of human cells [22,23] and the majority of CpG sites within the *MLH1* exons and introns were methylated. Together, these controls indicated that the NOMe-seq assays captured the true state of nucleosome positioning at *MLH1* splice sites.

Alternative splicing of *MLH1* occurs in a range of normal tissues [24], but several pieces of evidence indicate that the transcripts observed in our study are caused by the mutation, rather than being normal splicing events. First, the splicing aberrations were predicted with bioinformatic splicing software [13], and second, although one of the aberrant transcripts (Δ10) has been reported among naturally occurring *MLH1* splice transcripts, the clinical data associated with these variants are also indicative of pathogenicity as demonstrated by the results of previous multifactorial likelihood analyses [13].

One strength of our study was the ability to determine the allele-specific position of nucleosomes by incorporating a heterozygous single nucleotide variant into each NOMe-seq assay. Small changes in positioning were observed but these molecules constituted approximately the same small proportion of total molecules on both alleles. Subtle changes in nucleosome positioning have been considered important in regulating the expression of cell cycle-dependent genes [18] and enhancer accessibility [25], but in those studies the subtle changes were observed on a significant number of molecules that resulted in an overall change in nucleosome occupancy. There was no consistent alteration in nucleosome occupancy between the wild-type and mutant alleles at a variety of donor and acceptor sites, which would be expected if nucleosome positioning at these sites played a role in mediating the effects of splice site mutations. Together our data shows that alleles containing a splice site mutation show no differences in nucleosome occupancy to wild-type alleles.

A limitation of the approach was that in most of the assays (assays 2 to 6) it was only possible to measure nucleosome positions at either the donor site or the acceptor site. Bisulfite conversion causes fragmentation of genomic DNA and it is technically challenging to amplify fragments greater than 500 bp in length [26]. The donor and acceptor sites were separated by more than 2 kb of intronic sequence, making it impossible to concurrently determine the allele-specific position of nucleosomes at the donor and acceptor site on the same molecule. Although an independent NOMe-seq assay could be performed at the other donor or acceptor site, it would provide no information on allele-specificity. The data from one assay (c.588 + 1 G > T), however, in which both splice sites were present in the one amplicon, showed no significant change in nucleosome occupancy at the donor or acceptor site in intron 7.

Previous studies have utilised genome-wide datasets of micrococcal nuclease (MNase) digested chromatin to investigate nucleosome occupancy [3,4]. Here we used NOMe-seq to map the position of nucleosomes relative to exons and splice sites in a single gene at single molecule resolution. This approach provides the most accurate possible measurement of nucleosome positioning and enables allele-specific mapping of nucleosomes. In agreement with previous reports in humans [3,4], *Caenorhabditis elegans* [3,4] and *Drosophila melanogaster* [3], we observed high nucleosome occupancy at exons. Thus, the findings from genome-wide studies were supported by our independent, single-molecule approach at the *MLH1* gene.

A key finding of our study was that nucleosome positioning was not significantly affected by mutations at the *MLH1* splice sites investigated. Recent work with an *in vitro IKBKAP* mini-gene system found that alternative splicing changed chromatin organisation, with splice site strength and factors needed for splicing, such as U1 snSNP, playing a role in the regulation of nucleosome occupancy in exons [27]. This and an earlier study [3] indicate that splice site strength is an important determinant of nucleosome occupancy in exons. Exons with stronger polypyrimidine tracts (PPT; one of the conserved DNA elements located at 3′ splice sites) have increased nucleosome occupancy compared to immediately adjacent introns, suggesting that nucleosomes act as a barrier that help to define the physical location of the 3′ splice site [3]. Although we investigated an equal number of donor and acceptor splice site mutations, nucleosome positioning was not significantly different between exons and the immediate 5′ and 3′ intronic sequence on the wild-type or mutant allele. The discrepancy between earlier studies and ours may in part relate to the strength of the *MLH1* splice sites investigated. Alternatively, changes to chromatin organisation induced by splice mutations may be different to those caused by normal splicing events. Co-transcriptional splicing involves a complex interplay between RNAPII, the spliceosome [8] and histone modifications [3,9] and alterations in these factors, in addition to DNA sequence changes may be needed to alter nucleosome occupancy at exons.

In summary this study used NOMe-seq to determine the allele-specific position of nucleosomes in relation to *MLH1* splice site mutations. We conclude that splice site mutations that cause aberrant splicing of *MLH1* do not alone significantly affect local nucleosome positioning in LCLs from Lynch syndrome patients.
